# The cost-effectiveness of the use of selective media for the diagnosis of melioidosis in different settings

**DOI:** 10.1371/journal.pntd.0007598

**Published:** 2019-07-15

**Authors:** David A. B. Dance, Somsavanh Sihalath, Kolthida Rith, Amphone Sengdouangphachanh, Manophab Luangraj, Manivanh Vongsouvath, Paul N. Newton, Yoel Lubell, Paul Turner

**Affiliations:** 1 Lao-Oxford-Mahosot Hospital-Wellcome Trust Research Unit, Microbiology Laboratory, Mahosot Hospital, Vientiane, Lao PDR; 2 Centre for Tropical Medicine and Global Health, Nuffield Department of Medicine, Old Road Campus, University of Oxford, Oxford, England, United Kingdom; 3 Faculty of Infectious and Tropical Diseases, London School of Hygiene and Tropical Medicine, London, United Kingdom; 4 Cambodia Oxford Medical Research Unit, Angkor Hospital for Children, Siem Reap, Cambodia; 5 Mahidol-Oxford Tropical Medicine Research Unit, Bangkok, Thailand; University of Texas Medical Branch, UNITED STATES

## Abstract

**Background:**

Melioidosis is a frequently fatal disease requiring specific treatment. The yield of *Burkholderia pseudomallei* from sites with a normal flora is increased by culture using selective, differential media such as Ashdown’s agar and selective broth. However, since melioidosis mainly affects people in resource-poor countries, the cost effectiveness of selective culture has been questioned. We therefore retrospectively evaluated this in two laboratories in southeast Asia.

**Methodology/Principal findings:**

The results of all cultures in the microbiology laboratories of Mahosot Hospital, Vientiane, Laos and Angkor Hospital for Children, Siem Reap, Cambodia, in 2017 were reviewed. We identified patients with melioidosis who were only diagnosed as a result of culture of non-sterile sites and established the total number of such samples cultured using selective media and the associated costs in each laboratory. We then conducted a rudimentary cost-effectiveness analysis by determining the incremental cost-effectiveness ratio (ICER) per DALY averted and compared this against the 2017 GDP per capita in each country.

Overall, 29 patients in Vientiane and 9 in Siem Reap (20% and 16.9% of all culture-positive patients respectively) would not have been diagnosed without the use of selective media, the majority of whom (18 and 8 respectively) were diagnosed by throat swab culture. The cost per additional patient detected by selective culture was approximately $100 in Vientiane and $39 in Siem Reap. Despite the different patient populations (all ages in Vientiane vs. only children in Siem Reap) and testing strategies (all samples in Vientiane vs. based on clinical suspicion in Siem Reap), selective *B*. *pseudomallei* culture proved highly cost effective in both settings, with an ICER of ~$170 and ~$28 in Vientiane and Siem Reap, respectively.

**Conclusions/Significance:**

Selective culture for *B*. *pseudomallei* should be considered by all laboratories in melioidosis-endemic areas. However, the appropriate strategy for implementation should be decided locally.

## Author summary

Melioidosis is a frequently fatal disease caused by a soil bacterium called *Burkholderia pseudomallei*, that is widespread in the rural tropics. Because staff are often not familiar with it and because it may be hidden if it is outgrown by other bacteria, special culture media can help laboratories diagnose the disease. However, this costs more money so it is not always done even in areas where the disease is known to be present. We have looked at the results of a year’s bacterial cultures in two different laboratories in southeast Asia to identify how many patients were only identified using these special culture techniques, how much it cost, and whether the investment was considered worthwhile in terms of the gain in healthy life years in these patients who might otherwise have died had the disease not been diagnosed. Even though the laboratories adopted very different strategies for using the special media and served very different populations, in both places the use of the special techniques was very cost effective in terms not just of lives saved, but on purely financial grounds when compared with the GDP of each country.

## Introduction

*Burkholderia pseudomallei* is the causative agent of melioidosis, an important but under-recognised public health problem throughout the tropics [1]. It grows readily in clinical samples from normally sterile sites, such as blood cultures, aseptically aspirated pus from abscesses or body fluids. In sites with a normal flora, where it may be overgrown by other bacteria, the yield of culture can be improved by using selective differential media, such as Ashdown’s agar and broth, that suppress the growth of other organisms and encourage the formation of characteristic colonies [2, 3]. We have used these media routinely in our laboratories in melioidosis-endemic areas for more than 30 years, and have shown that they increase the number of cases of melioidosis diagnosed [3–11]. However, we have been fortunate to have research funding to support the associated costs of additional media, which would not be available to the majority of laboratories in endemic areas that have to make difficult decisions about the allocation of more limited resources. Melioidosis is unevenly distributed across endemic areas and others have questioned whether the routine use of selective media is cost effective [12, 13]. We therefore decided to review the cost-effectiveness of our current approach during 2017 in two different settings that used different strategies for the deployment of selective media for *B*. *pseudomallei* culture.

## Materials and methods

We reviewed the results of all cultures received during 2017 in the Microbiology laboratories of Mahosot Hospital, Vientiane, Lao People’s Democratic Republic and Angkor Hospital for Children, Siem Reap, Cambodia. We identified those patients with culture-confirmed melioidosis who were only detected by culturing specimens from non-sterile sites (throat swabs, wound and pus samples, sputum and endotracheal aspirates and urine) and then calculated the proportion of those who were only diagnosed through the use of selective media. Patients with positive blood or sterile body fluid cultures were excluded as these were, by definition, diagnosed without the need for selective media. Samples were classified according to whether *B*. *pseudomallei* was grown on non-selective media, on Ashdown’s agar (ASH) but not non-selective media, or from Ashdown’s selective enrichment broth subcultured onto Ashdown’s agar only (SB).

### Laboratory testing strategies

The testing strategies in the 2 hospitals were different.

In Vientiane, all throat swabs, pus and wound swabs, sputum and endotracheal aspirates, and urine samples received were cultured using selective media for *B*. *pseudomallei*. Throat swabs, which were sent specifically for *B*. *pseudomallei* culture from patients in whom melioidosis was suspected, were routinely cultured using selective media alone (one ASH plate and one SB) since previous experience has shown us that *B*. *pseudomallei* is rarely present in large numbers in such samples and is difficult to detect amongst the large numbers of colonies of other species that are invariably present on non-selective media [3]. Pus and wound swabs, sputum and endotracheal aspirates were cultured on ASH and SB in addition to non-selective media. The centrifuged deposit of urine samples was cultured on half an ASH plate in addition to routine semi-quantitative culture on chromogenic agar. All SB were subcultured onto half an ASH plate.

In Siem Reap, selective media were used only when the responsible clinician indicated a suspicion of melioidosis on the request form. Laboratory methods were similar to those used in Vientiane except that whole ASH plates were used for urine culture (half for neat urine and half for the centrifuged deposit) and SB subculture.

The consumable costs per Ashdown’s plate and broth were calculated for the additional selective media based on the prices of the ingredients in Thailand, where both laboratories purchase consumables and which was considered representative of melioidosis-endemic areas in Asia. These equated to 10 baht (approx. $0.31) per ASH and 8 baht (approx. $0.25) per SB.

### Identification of *B*. *pseudomallei*

In both laboratories, colonies suspected of being *B*. *pseudomallei* were screened by Gram stain, oxidase test and latex agglutination. Routine confirmation of identity was by API 20NE (bioMérieux, Basingstoke, UK).

### Cost-effectiveness analysis

We performed a rudimentary cost-effectiveness analysis as an indication as to whether the incremental costs of selective media would represent a judicious use of scarce resources in the two settings. The approach is similar to that used in a previous cost-effectiveness analysis of candidate melioidosis vaccines [14]. We considered the total additional costs for consumables for selective media and applied an additional 30 minutes of a microbiologist’s time per sample, costed at $2.90 per hour based on accounting records at the Cambodia site. We used an estimated 3% mortality rate in non-bacteraemic melioidosis patients when identified and treated, based on the literature [15] and our own data (mortality in non-bacteraemic cases 3.95% in Vientiane and 0.7% in Siem Reap), as compared with a mortality rate of 9% when undetected, based on the reported mortality before the introduction of modern treatment regimens [16]. We assumed that each death averted was associated with 66 Disability-Adjusted Life Years (DALYs) in Siem Reap, a paediatric hospital where the mean patient age was 5.7 years, and 37 DALYs in Vientiane where the mean patient age was 38.5 years, using the country-specific estimated life expectancy of individuals at this age [17]. The incremental cost-effectiveness ratio (ICER) per DALY averted was calculated by dividing the incremental costs of consumables and labour divided by the total number of DALYs averted (Eq 1).

$ICER=\frac{\left(cSM+cL\right)}{\Delta MR*YLL}$*Eq 1. ICER is the Incremental Cost Effectiveness Ratio, cSM is the incremental cost of selective media, cL are the incremental labour costs, ΔMR is the difference in mortality rates for patients with an untreated infection as compared with those that are treated, and YLL is the site-specific number of life years lost per death*.

The ICER was compared against the Laos and Cambodia 2017 GDP per capita ($2,457 and $1,384, respectively) to determine whether the use of selective media would be cost-effective.

### Ethics statement

All samples were submitted primarily for the purposes of routine diagnosis. In addition, many of the patients were included in studies of the aetiology of fever. In Cambodia the study was approved by the AHC Institutional Review Board (AHC IRB, 979–14) and the Oxford Tropical Research Ethics Committee (OxTREC 550–14). In Laos the study was approved by the Oxford Tropical Research Ethics Committee (OxTREC 006–07) and the Lao National Ethics Committee for Health Research (028–17). For these studies, patients or their parents or guardians provided written informed consent.

## Results

During 2017, 145 patients with culture-positive melioidosis were diagnosed in Vientiane, of whom 64 (44.1%) were only diagnosed by culture of 82 non-sterile sites. In Siem Reap, there were 53 cases of culture-positive melioidosis of whom 45 (84.9%) were only diagnosed by culture of 46 non-sterile sites (one patient was positive in superficial swab specimens from two anatomical sites). The breakdown of the positive samples and the media which were positive is shown in Table 1 and full results for individual culture-positive patients are given in S1 Table.

**Table 1:** Culture-positive samples and method by which positive.

Sample type	NSM	ASH	SB
**Throat swab (V)**	N/A	26	1
**Pus/wound swab (V)**	40	4	0
**Sputum/ET aspirate (V)**	3	4	1
**Urine (V)**	0	3	N/A
**Throat swab (SR)**	N/A	5	3
**Pus/wound swab (SR)**	37*	0	1

* 9 specimens were positive on non-selective plates and ASH/SB culture was not done. One specimen was positive on the non-selective plate and from subculture of the SB, but not on the primary ASH plate

Abbreviations: V, Vientiane; SR, Siem Reap; NSM, isolated on non-selective media; ASH, isolated by direct plating on Ashdown’s medium but not on non-selective media; SB, isolated by enrichment in Selective Broth and subculture on Ashdown’s medium only; N/A, not applicable; ET, endotracheal.

Eighteen patients in Vientiane and eight in Siem Reap were diagnosed by throat swab culture only, but most of these were positive on direct plating on ASH and only one positive throat swab in Vientiane and three in Siem Reap were detected by SB enrichment alone. The majority of pus samples and wound swabs were positive on non-selective media, with an additional four (9.1% of positives) detected through the use of ASH in Vientiane (with growth of small numbers of *B*. *pseudomallei* on ASH but no growth on non-selective plates) and none in Siem Reap, but the only additional yield from the use of SB for pus and wound swabs was from a single specimen in Siem Reap. Two of the specimens positive on ASH but not on non-selective media were swabs rather than pus and one was taken after several weeks of treatment. The numbers of positive sputum and endotracheal aspirates and urine samples were small and confined to Vientiane. More than half of the positive respiratory samples were only positive using selective media. Two patients in Vientiane were only diagnosed with melioidosis as a result of culture of a centrifuged deposit of urine on ASH.

Overall, in Vientiane 27 patients (18.6% of all culture-positive patients) would not have been diagnosed without the use of ASH, the majority of whom were only positive on throat swab culture, and an additional two (1.4%) would not have been diagnosed without the use of SB. In Siem Reap, five (9.4%) would not have been diagnosed without the use of ASH and four (7.5%) would not have been diagnosed without the use of SB.

During 2017 the Vientiane laboratory processed 2,130 throat swabs, 1,796 urines, 728 pus or wound swabs, 350 deep pus, 346 sputum and 142 ET aspirates. Table 2 shows the estimated consumable costs of the selective media used in processing these samples and the cost of detecting an additional case of melioidosis by using either ASH or SB on each sample type. The total consumable cost of using these additional selective media throughout the year was $2,921.02, meaning that the total consumables costs of each of the 29 additional cases detected using our current approach was almost exactly $100. The cost per additional positive sample detected varied considerably, from approximately $25 for an ASH plate on a throat swab to nearly $200 or more for the use of SB on each sample type.

**Table 2:** Additional yields and costs of selective culture by sample type.

Sample type		ASH				SB			
	Number of samples	Additional cost per sample ($)	Total cost ($)	Additional positives detected*	Cost per additional positive ($)	Additional cost per sample ($)	Total cost ($)	Additional positives detected	Cost per additional positive ($)
**Throat swab (V)**	2130	0.31	660.30	26	25.40	0.405	862.65	1	862.655
**Pus/wound swab (V)**	1078	0.31	334.18	4	83.55	0.405	436.59	0	∞
**Sputum/ET aspirate (V)**	488	0.31	151.28	4	37.82	0.405	197.64	1	197.64
**Urine (V)**	1796	0.155	278.38	3	92.79	N/A	N/A	N/A	N/A
**Throat swab (SR)**	122	0.31	37.82	5	7.56	0.56	68.32	3	22.77
**Pus/wound swab (SR)**	271	0.31	84.01	0	∞	0.56	151.76	1	151.76
**Sputum/ET aspirate (SR)**	1	0.31	0.31	0	∞	0.56	0.56	0	∞
**Urine (SR)**	22	0.31	6.82	0	∞	N/A	N/A	N/A	N/A

* Additional positives were patients with culture positive melioidosis who would not have been diagnosed without the use of selective media.

In Siem Reap, 416 samples were cultured during the year using selective media on the basis of clinical request at a consumables cost of $349.60, and this resulted in the diagnosis of nine cases of melioidosis that would not have been confirmed had selective media not been used, meaning that the consumable cost of selective culture per additional case detected was only approximately $39. The cost per additional positive detected was approximately $7.60 for culture of a throat swab on ASH and ranged between approximately $23 and $150 for the additional use of SB.

The additional 29 cases detected in Vientiane would corresponed with 64.4 DALYs averted, at a total cost of ~$10,966 including both consumables and labour, with an ICER of ~$170 per DALY averted. In Cambodia where only 416 samples were cultured with selective media the total cost was ~$903; the additional 9 detected cases would correspond with 31.9 DALYs averted and an ICER of ~$28; in both settings the use of selective media would therefore appear to be higly cost-effective, with an ICER well below the GDP per capita in their respective settings.

## Discussion

The additional yield from using selective media for culture of sites with a normal flora to detect patients with melioidosis is well established [3–9]. Overall the use of selective media in this study was responsible for the diagnosis of 20% of all the cases of melioidosis in Vientiane and 16.9% of the cases in Siem Reap, considerably more than in our previous study conducted in northeast Thailand nearly 30 years ago (3.5%) [3]. Since selective media only were used for throat swabs, it is not possible to compare the relative yields of selective and non-selective media, although in our previous studies *B*. *pseudomallei* grew on non-selective media from only 9 of 118 culture-positive throat throat swabs [3]. However, the relative benefit of using selective media will depend on several factors such as the local incidence of melioidosis, local clinical practice, the laboratory testing strategy adopted, and the cost of the media themselves. Understanding the local costs and benefits of different approaches is clearly of importance to laboratory managers in melioidosis-endemic areas who have to make decisions about the most effective way to deploy scarce resources in order to bring about the greatest benefit to patients. This question came up repeatedly during a recent workshop on melioidosis in Cambodia [18], prompting us to undertake the current analysis. While further context-specific analyses are advised, our findings from two different settings indicate that more extensive use of selective media than is commonly practiced in many endemic areas is likely to be highly cost-effective.

There has been one previous attempt to estimate the cost-effectiveness of selective culture for melioidosis diagnosis [13]. This study, which took place in Kuala Lumpur, Malaysia, an area of relatively low melioidosis incidence, and predominantly entailed respiratory samples, estimated the costs to detect one additional culture and patient as $25 and $75, respectively. Another study in Kampong Cham Provincial Hospital, Cambodia found that only one additional *B*. *pseudomallei* positive sample was identified amongst 241 sputum samples cultured using SB and ASH. Clearly the local incidence of melioidosis is likely to be one of the factors with the greatest impact on the cost effectiveness of selective culture. However, the testing strategy adopted (e.g. using selective culture on all samples of particular types as opposed to culturing only those from patients in whom clinicians indicate a suspicion of melioidosis) will also have a major impact on cost-effectiveness.

Overall, in our study the costs of selective media per additional case detected varied considerably between the two laboratories as a result of the difference in screening strategy. In Vientiane, the cost of selective media using the current approach was approximately $380 per additional case diagnosed (including both consumables and labour) as opposed to only $100 in Siem Reap. The relative benefits of adding SB enrichment also varied between the sites, with only 2 of 39 samples that were positive by selective culture alone in Vientiane being detected through broth enrichment as opposed to 4 of 9 in Siem Reap. Clearly there are differences between the patient populations in the two centres, with patients in Vientiane being from all age groups whereas only children were included in Siem Reap, which might have affected the quality of the samples collected. While this emphasises the fact that decisions about the most appropriate culture strategy needs to be decided at a local level, the very low ICERs imply that more extensive use of selective media could cost-effectively yield additional gains. This is particularly likely in Cambodia, where the use of selective media was restricted to patients in whom the clinician suspected melioidosis. Assuming a willingness to pay $1,384 per DALY averted (the Cambodia GDP per capita), spending up to $50 per patient on selective media cultures in other patients would remain cost-effective even if the prevalence of undetected cases in these patients was as low as 1%.

In both sites, culture of a throat swab on ASH was confirmed as an effective strategy for detecting patients with melioidosis who were not diagnosed in other ways. In Vientiane, the majority of throat swabs are submitted specifically for the investigation of melioidosis. This does not reflect the presence of pharyngitis in most of these patients, but is likely mainly to represent contamination of the throat by lower respiratory tract secretions. It is not common practice in Laos for clinicians to send sputum for bacteriological culture. Had this been the case, the additional benefit from culture of throat swabs is likely to have been less. However, culture of sputum and ET aspirates on ASH was also relatively cost-effective in Vientiane. The additional benefit from using ASH for culture of pus/wound swabs and urine samples was less than that for throat swabs, and therefore correspondingly less cost-effective, but both methods did detect some patients with melioidosis who would not have been diagnosed in other ways. The use of ASH for culture of pus from previously undrained abscesses is unlikely to be worthwhile, as in melioidosis this is likely to grow pure *B*. *pseudomallei*, although occasional dual infections (e.g. with *Staphylococcus aureus*) can occur. However, the use of ASH is more likely to be of benefit in superficial wound swabs and abscesses that have already ruptured, as these may be colonised with a range of flora that could outgrow *B*. *pseudomallei* on non-selective media, or in patients on treatment. We consider that the culture of the centrifuged deposit of a urine sample on ASH is also worthwhile in all patients suspected of having melioidosis, as in some patients this may be the only positive sample, and the number of organisms present is often well below the threshold for ‘significant bacteriuria’ with other pathogens, even in patients with prostatic involvement [6].

There are several approaches that might be used to reduce the costs associated with selective culture to detect *B*. *pseudomallei*. These include the omission of SB enrichment for some or all samples, the use of half rather than whole plates, the use of cheaper media, and the testing of only selected specimens rather than all specimens of a particular type. Several other media have been recommended for the selective isolation of *B*. *pseudomallei* from clinical samples [7, 19–23]. Our study has focused only on the use of ASH and SB, which were the media in use in our laboratories during 2017. Studies comparing *B*. *pseudomallei* selective media on clinical samples are relatively rare and further comparative evaluations of different formulations would be useful, although it is unlikely that any other media would be significantly cheaper than ASH and SB. One issue of particular concern is that ASH relies on gentamicin to suppress Gram negative organisms and as the prevalence of antimicrobial resistance increases it may become less effective and new formulations may become necessary [23]. The use of half plates is worthy of consideration, although this can make colonies of *B*. *pseudomallei* more difficult to pick out from other local flora.

In conclusion, this analysis has caused us to review our own approach to the use of selective media for the diagnosis of melioidosis. In any patients with suspected melioidosis we recommend that, in addition to blood culture, a throat swab, or a good quality sputum sample if available, should be sent specifically for culture on ASH and in SB, and a centrifuged deposit of urine should also be cultured on ASH. In areas of high melioidosis incidence, we also recommend the use of ASH and SB for any sputum and endotracheal aspirates received from patients with pneumonia. However, we do not consider that the routine use of selective media for culture of pus samples and wound swabs is warranted unless the request form specifically requests investigation for melioidosis. Others must decide on the appropriate way to deploy selective media according to their local epidemiology, clinical practice and available resources.

## Supporting information

S1 Table Results of culture on different media from Laos and Cambodia by patient and sample type. (XLSX)
